# Bone morphogenetic protein 7 sensitizes O6-methylguanine methyltransferase expressing-glioblastoma stem cells to clinically relevant dose of temozolomide

**DOI:** 10.1186/s12943-015-0459-1

**Published:** 2015-11-06

**Authors:** Jonathan L. Tso, Shuai Yang, Jimmy C. Menjivar, Kazunari Yamada, Yibei Zhang, Irene Hong, Yvonne Bui, Alexandra Stream, William H. McBride, Linda M. Liau, Stanley F. Nelson, Timothy F. Cloughesy, William H. Yong, Albert Lai, Cho-Lea Tso

**Affiliations:** Department of Surgery/Surgical Oncology, David Geffen School of Medicine, University of California at Los Angeles, Los Angeles, CA USA; Department of Neurosurgery, Guangzhou General Hospital of Guangzhou Military Command, Guangzhou, Guangdong China; Department of Advanced Molecular and Cell Therapy, Kyushu University Hospital, Higashiku, Fukuoka Japan; Department of Orthopedics, Zhongshan Hospital, Xiamen University, Xiamen, China; Department of Radiation-Oncology, David Geffen School of Medicine, University of California Los Angeles, Los Angeles, CA USA; Department of Neurosurgery, David Geffen School of Medicine, University of California at Los Angeles, Los Angeles, CA USA; Department of Human Genetics, David Geffen School of Medicine, University of California at Los Angeles, Los Angeles, CA USA; Department of Neurology, David Geffen School of Medicine, University of California at Los Angeles, Los Angeles, CA USA; Department of Pathology and Laboratory Medicine, David Geffen School of Medicine, University of California at Los Angeles, Los Angeles, CA USA; Jonsson Comprehensive Cancer Center, University of California at Los Angeles, Los Angeles, USA

**Keywords:** BMP7, Glioblastoma, Temozolomide, MGMT, Glioblastoma stem cells

## Abstract

**Background:**

Temozolomide (TMZ) is an oral DNA-alkylating agent used for treating patients with glioblastoma. However, therapeutic benefits of TMZ can be compromised by the expression of O6-methylguanine methyltransferase (MGMT) in tumor tissue. Here we used MGMT-expressing glioblastoma stem cells (GSC) lines as a model for investigating the molecular mechanism underlying TMZ resistance, while aiming to explore a new treatment strategy designed to possibly overcome resistance to the clinically relevant dose of TMZ (35 μM).

**Methods:**

MGMT-expressing GSC cultures are resistant to TMZ, and IC50 (half maximal inhibitory concentration) is estimated at around 500 μM. Clonogenic GSC surviving 500 μM TMZ (GSC-500 μM TMZ), were isolated. Molecular signatures were identified via comparative analysis of expression microarray against parental GSC (GSC-parental). The recombinant protein of top downregulated signature was used as a single agent or in combination with TMZ, for evaluating therapeutic effects of treatment of GSC.

**Results:**

The molecular signatures characterized an activation of protective stress responses in GSC-500 μM TMZ, mainly including biotransformation/detoxification of xenobiotics, blocked endoplasmic reticulum stress-mediated apoptosis, epithelial-to-mesenchymal transition (EMT), and inhibited growth/differentiation. Bone morphogenetic protein 7 (BMP7) was identified as the top down-regulated gene in GSC-500 μM TMZ. Although augmenting BMP7 signaling in GSC by exogenous BMP7 treatment did not effectively stop GSC growth, it markedly sensitized both GSC-500 μM TMZ and GSC-parental to 35 μM TMZ treatment, leading to loss of self-renewal and migration capacity. BMP7 treatment induced senescence of GSC cultures and suppressed mRNA expression of CD133, MGMT, and ATP-binding cassette drug efflux transporters (ABCB1, ABCG2), as well as reconfigured transcriptional profiles in GSC by downregulating genes associated with EMT/migration/invasion, stemness, inflammation/immune response, and cell proliferation/tumorigenesis. BMP7 treatment significantly prolonged survival time of animals intracranially inoculated with GSC when compared to those untreated or treated with TMZ alone (*p* = 0.0017), whereas combination of two agents further extended animal survival compared to BMP7 alone (*p* = 0.0489).

**Conclusions:**

These data support the view that reduced endogenous BMP7 expression/signaling in GSC may contribute to maintained stemness, EMT, and chemoresistant phenotype, suggesting that BMP7 treatment may provide a novel strategy in combination with TMZ for an effective treatment of glioblastoma exhibiting unmethylated MGMT.

**Electronic supplementary material:**

The online version of this article (doi:10.1186/s12943-015-0459-1) contains supplementary material, which is available to authorized users.

## Background

Glioblastoma is the most common and lethal primary malignant brain tumor which remains a challenging disease to treat. The current standard-of-care for patients with newly diagnosed glioblastoma consists of maximal surgical resection, radiotherapy (RT), and concomitant and adjuvant chemotherapy with temozolomide (TMZ). Despite aggressive treatment, all patients eventually suffer from tumor progression because their tumors become resistant to maintenance TMZ, and the median survival among all patients is only 12–15 months from diagnosis. Although TMZ is the principal first-line chemotherapeutic agent used for the treatment of glioblastoma, it does not significantly prolong the overall survival of patients without methylation of the MGMT promoter [1–3]. The anti-tumor activity of TMZ depends on its ability to methylate DNA at the O-6 positions of guanine residues, which will produce methylguanine adducts, triggering a continuous cycle of DNA base mismatch repair, which leads to double-strand breaks and base mispairing, ultimately inducing cell apoptosis [4, 5]. MGMT is a cellular DNA repair protein that neutralizes the cytotoxic effects of TMZ by directly transferring methyl groups from the O-6-position of guanine to a cysteine residue [6]. Therefore, glioblastoma tumors expressing MGMT have been implicated as a major intrinsic mechanism of resistance to TMZ, although a different mechanism independent of MGMT has been reported [7]. Methylation of MGMT promoter has become an important prognostic and predictive factor for TMZ treatment of newly diagnosed GBM, and high MGMT protein expression in patient tumors is associated with TMZ resistance in patients [8, 9]. Thus, treatment strategies to overcome MGMT-dependent or independent chemoresistance are urgently needed.

It has been hypothesized that glioblastoma stem cells (GSC) are responsible for post-treatment tumor recurrence because they are drug-resistant cells that can survive treatment and regenerate tumors [10–13]. A recent study using a genetically engineered mouse model of glioma has provided direct evidence and demonstrated that a quiescent subset of endogenous stem-like glioma cells is located at the apex of a cellular hierarchy in tumor maintenance, and is responsible for tumor recurrence after TMZ therapy fails [14]. This study thus supports the view that TMZ can only deplete the proliferative differentiated tumor population, but not the quiescent GSC. It is generally accepted that GSC are a small subset of slow-cycling stem-like glioblastoma tumor cells within a tumor tissue, that are capable of clonally self-renewing and growing as tumor spheres and migrating radially outward in culture, and reconstituting a tumor in mouse brain that recapitulates the histopathological features of the patient tumor from which the GSC were derived [15–17]. A previous study indicated that MGMT-negative CSC line can be depleted with 50 μM TMZ treatment in culture whereas GSC line expressing MGMT transcripts results in a 10-fold increase of TMZ-resistance (500 μM) [18]. Similarly, we also found that GSC clones resistant to radiochemotherapy expressed upregulated MGMT when compared to that of autologous sensitive GSC clones [13]. Current adjuvant TMZ treatment is given as 150–200 mg/m^2^ on days 1 to 5 of a 28-day cycle, which results in concentrations of between 15–35 μM in glioma tumor tissue [19]. Therefore, identifying a new strategy to sensitize the MGMT-expressing GSC to clinically achievable dose of TMZ in brain will have important implications for the management of glioblastoma patients with unmethylated MGMT promoter.

In this study, we extended our previous work and used MGMT-expressing GSC that survived 500 μM TMZ treatment (GSC-500 μM TMZ) to explore the potential intrinsic factors that may be linked to triggering TMZ resistance. By comparing gene-expression profiles between GSC-500 μM TMZ and parental GSC (GSC-parental), we explored a series of genes that characterized intracellular stress responses and self-defense mechanisms against high-dose TMZ in GSC-500 μM TMZ. Moreover, BMP7 was identified as a top down-regulated gene in GSC-500 μM TMZ. We thus evaluated the treatment efficacy of recombinant BMP7 on GSC and tested the synergistic effect of BMP7 and low-dose TMZ on the treatment of MGMT-expressing GSC both *in vitro* and *in vivo*. We further investigated the potential MGMT-independent mechanisms contributing to the BMP7-mediated sensitization of MGMT-expressing GSC to TMZ.

## Results

### GSC cultures contain tumorigenic clones that are resistant to high-dose TMZ and express upregulated MGMT

**Fig. 1:**
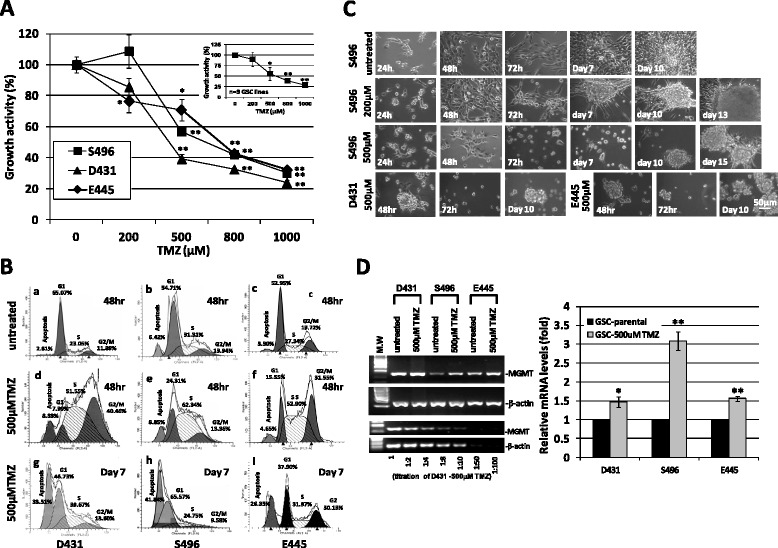
A selection of clonogenic GSC clones able to survive high-dose TMZ treatment from MGMT-expressing GSC culture lines. **a**. Growth activity of GSC lines treated with indicated TMZ doses was determined via MTS-based cell proliferation assay. The dose response curve of GSCs derived from each patient tumor, is presented both individually and combined together. The concentration of TMZ required for 50 % inhibition of GSC viability *in vitro* (IC50) was estimated using the mean of growth activity of 3 GSC lines. Values of TMZ-treated cells represent the percentage of growth relative to that of untreated cells, which was converted to 100 %. Data represent mean values ± SD of triplicate measurements of three independent experiments. *p < 0.05 and **p < 0.001 in relation to untreated control. **b**. Cell cycle distribution of GSC treated with and without 500 μM TMZ was determined on day 2 or day 7 after treatment. Numbers in the respective cell cycle distributions indicate the percentage of cells in each phase of the cell cycle. **c**. *In vitro* selection of clonogenic survival of GSC in the presence of 200 or 500 μM TMZ or left untreated. Photos were taken at indicated time periods after treatment. **d**. sqRT-PCR analysis of MGMT mRNA expression levels in untreated parental GSC (GSC-parental) and clonogenic clones surviving 500 μM TMZ treatment (GSC-500 μM TMZ). The graph shows the mean values of fold change for MGMT mRNA expression levels in indicated GSC-500 μM TMZ lines relative to those of untreated GSC-parental. All values are relative to those of the internal control gene β-actin, with values of GSC-500 μM TMZ representing the fold change relative to that of GSC-parental, which was converted to 1. Data represent mean values ± SD of triplicate measurements in three independent experiments. *p < 0.05 and **p < 0.001 in relation to GSC-parental

**Fig. 2:**
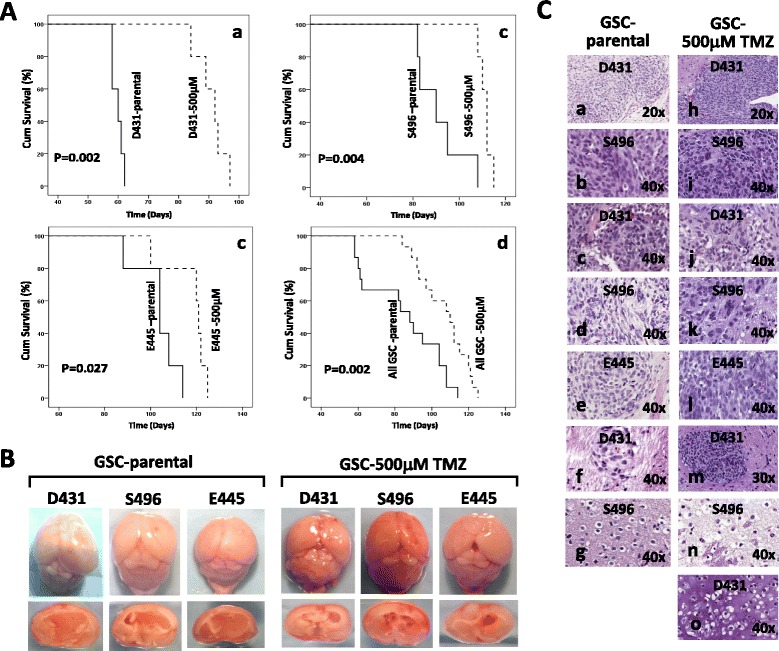
Clonogenic clones surviving 500 μM TMZ treatment showed a delay in tumor formation compared to those of unselected parental GSC. **a**. Kaplan-Meier survival curves of indicated GSC-parental (5 mice/group) and GSC-500 μM TMZ (5 mice/group). *P*-values were calculated using the Log-rank test. The survival curves were plotted for individual (*a*–*c*) and combined 3 GSC (*d*). **b**. Representative macrophotographic image of glioma xenografts initiated by GSC-parental and GSC-500 μM TMZ that are growing in the intracranial site. **c**. Representative hematoxylin and eosin (HE) staining of xenograft tumors. Brain tissues from mice injected with either GSC-parental (D431, S496, E445) or GSC-500 μM TMZ (D431, S496, E445) display invasive growth of gliomas and exhibit histopathological features of human glioblastoma, including hypercellularity (*a*, *h*), hyperchromatism (*b*, *i*), pleomorphism and mitosis (*c*–*e*, *j*–*l*), vascular endothelial hyperplasia (*f*, *m*), oligodendroglial components (*g*, *n*), and chondrocytic components (*o*)

Three previously established and characterized patient tumor-derived MGMT unmethylated GSC cell lines, D431, S496 and E445 [13, 17, 20] were used to study TMZ sensitivity. A cell viability assay was performed to determine the dose-dependent effect and IC50 of TMZ for GSCs (Fig. 1a). Cells were plated at clonal density and treated with various doses of TMZ (0, 200, 500, 800, 1000 μM) and incubated for 48 h prior to measurement of their growth activity. All three GSC lines are relatively resistant to 200 μM TMZ, but showed a dose response to higher-dose TMZ treatment (Fig. 1a); the IC50 value of TMZ against three tested GSC lines is found to be approximately 500 μM. To understand how high-dose TMZ affects the growth activity of GSC, we compared their cell-cycle distributions in their normal untreated state, and after both 48-hour and 7-day exposure to TMZ. The cell cycle analysis at 48-hr time point of 500 μM TMZ treatment revealed a robust cell cycle arrest in the S phase for all 3 tested GSC (D431/23 %, S496/31 %, E445/27 % Vs. 52 %, 62 %, 53 % respectively), whereas arrest in G2/M phase was only observed in 2 cultures (12 %, 14 %, 20 % Vs. 40 %, 13 %, 32 % respectively) (Fig. 1b, *a*–*f*). TMZ-induced cellular damage was clearly seen on day-7 cultures and resulted in a sub-G1 peak where 26–42 % of apoptotic cells were determined (Fig. 1b, *g*–*i*). Meanwhile, several clonogenic cells survived and started to re-populate tumor spheres, and large tumor spheres can be seen on day 10 for all three cultures (Fig. 1c). When compared to untreated, parental GSC, these clonogenic GSC surviving 500 μM TMZ, designated as GSC-500 μM TMZ, expressed upregulated DNA repair enzyme MGMT mRNA as determined by semi-quantitative RT-PCR (sqRT-PCR) (Fig. 1d). To test whether GSC-500 μM TMZ possess tumorigenic capacity, cells were stereotactically injected into the brains of severe combined immunodeficient (SCID) mice. Mice which received cells derived from GSC-parental (5 mice per GSC line) or GSC-500 μM TMZ (5 mice per GSC line) all developed tumors, while delayed tumor development was determined in all 3 GSC-500 μM TMZ lines (Fig. 2a, b). The H-E staining of xenograft tumors initiated by GSC-parental or GSC-500 μM TMZ showed no histological difference between the two groups, indicating that *in vitro* selection by high-dose TMZ did not alter/destroy the properties and histological origin of GSC. All tumors demonstrated invasive growth of gliomas with diffuse infiltration into the surrounding tissue and vessels, and recapitulated the typical histopathological features of human glioblastoma (Fig. 2c). These data indicated that MGMT-expressing GSC-parental cultures contain minor stem-like tumor-initiating cells with inherent properties that allow them to adapt to deadly stress induced by high-dose TMZ.

### Molecular profiles of GSC-500 μM TMZ revealed an intrinsic defense strategy against high- dose TMZ

**Fig. 3:**
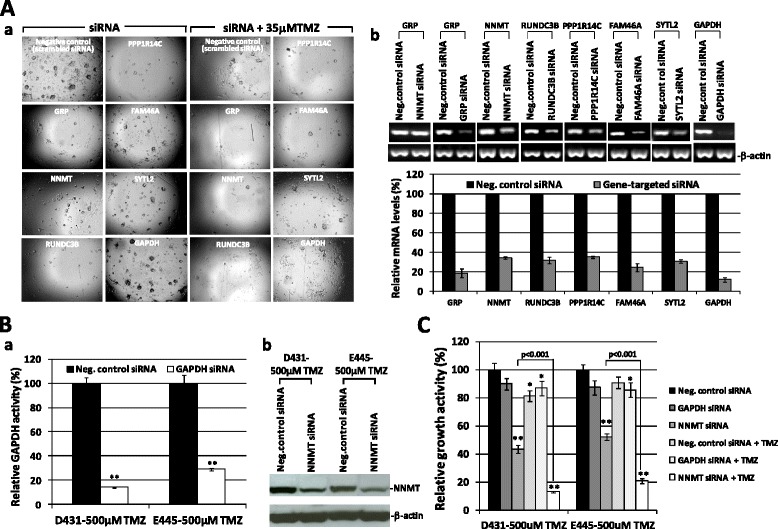
Inhibition of GSC self-renewing capacity by knockdown of selected defense signatures of GSC-500 μM TMZ. **a**. GSC-500 μM TMZ were treated with siRNA targeting indicated defense signatures of GSC-500 μM TMZ in the presence or absence of 35 μM TMZ. Representative photos (D431-500 μM TMZ) were taken 7 days after treatment (*a*). On-target gene knockdown by siRNA treatment was verified by sqRT-PCR analysis 48 h after transfection (*b*). The graph shows the mean values of mRNA expression levels of siRNA-targeted genes in GSC-500 μM TMZ lines relative to those of treated with negative control siRNA. All values were relative to those of the internal control gene β-actin, with values of GSC-500 μM TMZ representing the percentage of mRNA expression levels relative to that after treatment with negative control siRNA, which was converted to 100 %. Data represent mean values ± SD of triplicate measurements in three independent experiments in two GSC lines (D431-500 μM TMZ, E445-500 μM TMZ). All gene-targeted values represent statistically significant reduction of mRNA levels (*P* < 0.001). **b**. Gene knockdown verification on the protein level. The siRNA-mediated knockdown of GAPDH and NNMT expressions in GSC at protein levels was determined by measurement of GAPDH enzymatic activity (*a*) and Western blot analysis (*b*), respectively. Data represent mean values ± SD of triplicate measurements in three independent experiments. **p < 0.001 in relation to treatment with negative control siRNA. **c**. The effects of gene knockdown on GSC self-renewing capacity. The GSC growth under the indicated treatment conditions was determined by MTS assay, which was carried out 72 h after transfection. Data represent mean values ± SD of triplicate measurements in three independent experiments. *p < 0.05 and **p < 0.001 in relation to cells treated with negative control siRNA

To explore the intrinsic mechanisms allowing clonogenic clones to overcome or adapt to 500 μM TMZ, we performed a comparative high-density expression microarray analysis of GSC-parental (*n* = 3 patients, duplicate/6 samples) and GSC-500 μM TMZ survived one and two-cycle 500 μM TMZ treatment (*n* = 3 patients, 6 samples). Probe set signals on the expression array that were **≥** 1.5-fold higher or lower in GSC-500 μM TMZ group versus the GSC-parental group with a pairwise t-test (*P* < 0.05) were selected, and 36 informative genes were obtained (Table 1). As anticipated, the overall gene expression profile revealed the activation of stress response pathways for self-defense and stabilizing cellular/genomic integrity, including blocking of endoplasmic reticulum stress-mediated apoptosis (GRP), biotransformation of xenobiotics/detoxification (NNMT), inhibition of insulin/Akt signaling (EGR1, INPP4B), induction of cellular quiescence/growth inhibition (CDKN1A, NR2F1, SLFN5. PPP1R14C, ZNF652, PRRG4), stabilization of mitochondria/microtubule network (MAP4), promoting EMT, migration/invasion (RUNDC3B, MCAM, NR2F1, RRAS, FN1, MALAT1), angiogenesis (GRP, APOLD1, FN1, MALAT1), and suppression of cell proliferation and differentiation (downregulated BMP7, FJX1). The distinct gene expression in GSC-parental and GSC-500 μM TMZ was confirmed by sqRT-PCR analysis (Additional file 1: Figure S1). To test whether upregulated “defense signatures” indeed provide protection from TMZ treatment, cells were treated with siRNA targeting selected defense signatures for 48 h prior to receiving 35 μM TMZ. Active clonogenic growth was detected in cells treated with siRNA negative control with or without addition of 35 μM TMZ even though less growth was seen in TMZ-treated wells, the on-target knockdown of selected defense signatures have resulted in a severe loss of ability to repopulate tumor spheres (Fig. 3a, *a*; Additional file 1: Figure S2). Moreover, adding 35 μM TMZ after knockdown of defense genes further lysed tumor sphere-initiating cells (Fig. 3a, *a*; Additional file 1: Figure S2), which was not seen in those transfected with siRNA targeting housekeeping gene, glyceraldehyde-3-Phosphate Dehydrogenase (GAPDH) (Fig. 3a, *a*). On-target gene knockdown by siRNA treatment was verified by sqRT-PCR analysis (Fig. 3a, *b*) and gene knockdown at protein levels was validated in two representative genes, using Western blot analysis (NNMT) and enzyme assay (GAPDH) (Fig. 3b). The differential growth effect of siRNA treatment on GSC was further validated by cell proliferation assay and a TMZ-specific synergy was demonstrated in GSC knockdown of defense signature, not GAPDH (*p* < 0.001) (Fig. 3c). These results suggest that some of the upregulated genes in GSC-500 μM TMZ not only serve as essential factors for maintaining GSC integrity, but also play a key role in maintaining resistance against lethal stress induced by high-dose TMZ.

**Table 1:** Molecular signatures and defense profiles of glioblastoma stem cells (GSC) resistant to 500μM TMZ^a^

Gene	Fold change	*P* value	Functional involvement
MB2: metastasis related protein	9.48	0.037251	human lung cancer cell metastasis-related gene
GRP: gastrin-releasing peptide	3.03	0.012739	activates stress responses; blocks ER stress-mediated apoptosis; angiogenesis
NNMT: nicotinamide N-methyltransferase	3.02	0.016864	biotransformation of xenobiotics, detoxification, drug resistance, DNA repair
RUNDC3B: RUN domain containing 3B	2.63	0.022848	tumor invasion, tumorigenic capacity, malignant transformation
PPP1R14C: protein phosphatase 1, regulatory (inhibitor) subunit 14C	2.45	0.038521	tumor suppressor, upregulates early growth response 1 and PTEN gene expression
FAM46A: family with sequence similarity 46, member A	2.35	0.026347	retinal signaling pathways
SYTL2: synaptotagmin-like 2	2.07	0.006201	vesicle trafficking
EGR1: early growth response 1	2.00	0.012287	impaired insulin/Akt signaling, reduced glucose uptake, autophagy, Sirt1 expression
ZNF652: zinc finger protein 652	1.88	0.024023	a transcriptional repressor, tumor suppressor
MAP4: microtubule-associated protein 4	1.85	0.007016	stabilizes mitochondria, microtubule network, and cell viability
APOLD1: apolipoprotein L domain containing 1	1.85	0.006038	angiogenesis, blood-brain permeability
ANKRD10: ankyrin repeat domain 10	1.85	0.011112	unknown
C5orf32: chromosome 5 open reading frame 32	1.79	0.005850	stress tolerance
SLFN5: Schlafen family member 5	1.74	0.025479	growth-inhibitory responses, tumor suppressor
MCAM: melanoma cell adhesion molecule	1.73	0.004659	cell adhesion, EMT
MST150: MSTP150	1.72	0.010533	induced by nerve growth factor
NR2F1: Nuclear receptor subfamily 2, group F, member 1	1.68	0.003457	tumor dormancy, cell motility, and invasiveness
PRRG4: Proline rich Gla (G-carboxyglutamic acid) 4 (transmembrane)	1.65	0.009662	downregulates ERK 1/2 signaling, cell cycle control
RRAS: related RAS viral (r-ras) oncogene homolog	1.62	0.022906	maintains endothelial barrier function, cell migration
CDKN1A: cyclin-dependent kinase inhibitor 1A (p21, Cip1)	1.60	0.018892	cell cycle regulator, cellular quiescence, tumor suppressor
FAM114A1: family with sequence similarity 114, member A1	1.59	0.026679	neuronal cell development
INPP4B: inositol polyphosphate-4-phosphatase, type II, 105kDa	1.57	0.004296	phospholipid metabolism, tumor suppressor, inhibits PI3K/Akt signaling
FN1: fibronectin 1	1.56	0.037537	EMT, cell adhesion and migration, angiogenesis
C6orf57: chromosome 6 open reading frame 57	1.54	0.036557	mitochondrial function
VDAC1: voltage-dependent anion channel 1	1.52	0.005105	respiratory electron transport
PSAT1: phosphoserine aminotransferase 1	1.52	0.029482	serine synthesis pathway; amino acid/phospholipid/nucleotide synthesis
ZEB1: zinc finger E-box binding homeobox 1	1.51	0.038506	transcriptional repression of interleukin 2
MALAT1: metastasis associated lung adenocarcinoma transcript 1	1.51	0.021777	EMT, migration, invasion, metastasis, angiogenesis
FAT3: FAT tumor suppressor homolog 3 (Drosophila)	1.51	0.014515	controls neuronal morphology
MFSD2: major facilitator superfamily domain containing 2	−1.53	0.006302	plays a role during fasting and adaptive thermogenesis
NUP160: nucleoporin 160kDa NUP98: nucleoporin 98kDa	−1.55	0.049580	promotes mitotic spindle assembly, contributes to proper kinetochore functions
NUP98: nucleoporin 98kDa	−1.59	0.032310	a nuclear pore complex component; a transcription factor
DPP6: dipeptidyl-peptidase 6	−1.06	0.013619	interaction with extracellular matrix
FJX1: four jointed box 1 (Drosophila)	−1.62	0.004725	growth and differentiation
NRP2: neuropilin 2	−1.63	0.041599	cardiovascular development, axon guidance, and tumorigenesis
BMP7: bone morphogenetic protein 7 (osteogenic protein 1)	−1.98	0.002699	cartilage and bone formation; differentiation and proliferation

*ER* endoplasmic reticulum; *EMT* epithelial-to-mesenchymal transition

^a^Probe set signals on the expression array that were ≥ 1.5-fold different in GSC-500 μM TMZ (*n*=3 patients, 6 samples) when compared to GSC-parental (*n* = 3 patients, duplicate samples) by a pairwise t-test (*P* < 0.05), were selected. Samples were permutated 100 times by dChip, and 36 annotated genes with median FDR = 4 % were obtained

### BMP7 treatment reverses chemoresistance of MGMT-expressing GSC to TMZ

**Fig. 4:**
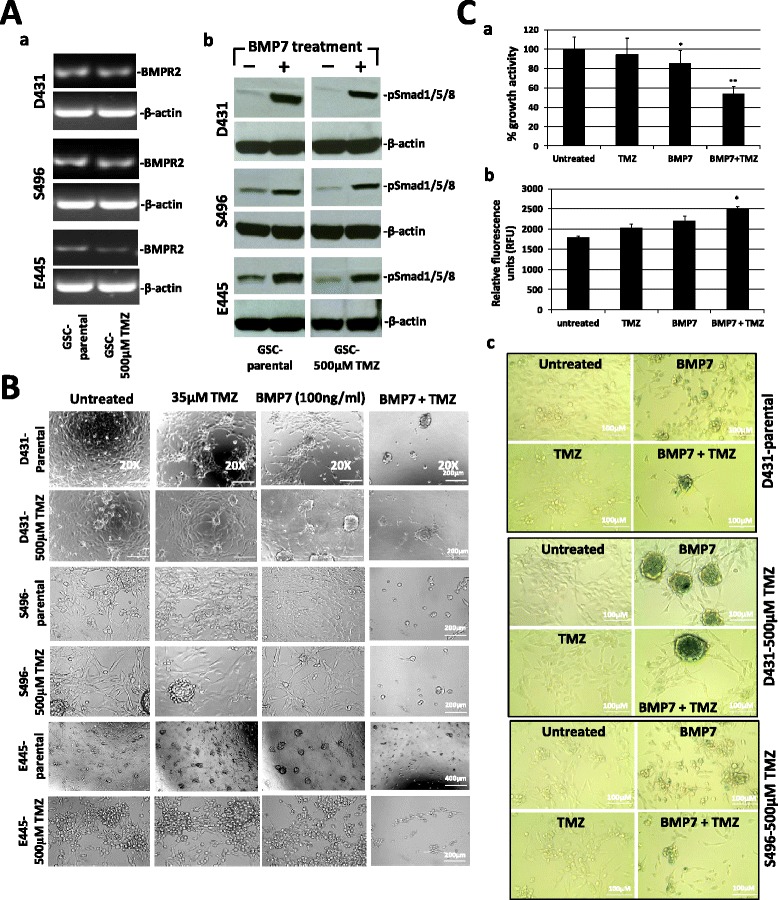
Sensitization of MGMT-expressing GSC to low-dose TMZ treatment by recombinant BMP7. **a**. The mRNA expression of bone morphogenetic protein receptor, type II (serine/threonine kinase) (BMPRII) in GSC was verified by sqRT-PCR analysis (*a*) and BMP7-induced Smad1/5/8 phosphorylation in GSC was assayed by Western blot analysis (*b*). **b**. GSC-parental and GSC-500 μM TMZ were treated with 35 μM TMZ, BMP7 (100 ng/ml) or combination of BMP and TMZ. Treatment procedures were described in the text. Representative photos were taken 6–8 days after treatment. **c**. The growth effects (*a*), cell apoptosis (*b*), and cell senescence (*c*) induced by the indicated treatments on GSC were determined by a cell proliferation assay, the measurement of Caspase3/7 activity, and senescence-associated beta-galactosidase staining respectively. Blue staining in cells is indicative of cellular senescence (*c*). Data in (*a*) and (*b*) represent mean values ± SD of triplicate measurements in three independent experiments. *p < 0.05 and **p < 0.001 in relation to untreated GSC

BMP7 was identified as the top down-regulated gene in GSC-500 μM TMZ by expression microarray analysis when compared to autologous GSC-parental and suggested the endogenous BMP7 expression in GSC may play a role in regulating drug resistance to TMZ. We therefore, focused our study on BMP7 for the following reasons: recombinant BMP7 is readily available, it can cross the blood-brain barrier (BBB) [21], and it has been regarded as a metastatic suppressor gene. Since all tested GSC cultures expressed BMP type 2 receptors (BMPR2) (Fig. 4a, *a*), and BMP7 treatment induced phosphorylation of Smad1/5/8 protein (Fig. 4a, *b*), we hypothesized that augmenting BMP7 signaling in GSC would attenuate stemness and EMT properties, and sensitize cells to TMZ treatment via switching off defense genes. To test this hypothesis, both GSC-parental and GSC-500 μM TMZ were seeded and either treated with TMZ (35 μM), BMP7 (100 ng/ml), combination of BMP7 and TMZ, or left untreated. In the combination treatment, GSC were pre-treated with BMP7 for overnight, and again on the following day by adding BMP7 to cultures for 30 min prior to TMZ treatment. All treatments were repeated for 5 consecutive days. GSC treated with 35 μM TMZ only showed minimal growth effect when compared to untreated GSC. Although GSC treated with BMP7 showed less cell migration and proliferation when compared to untreated GSC, no cell death/lysis was observed in cultures, and cells continued to grow after treatment (Fig. 4b). In contrast, combination treatment with BMP7 and TMZ showed a progressive suppression of both cell proliferation and migration starting on day 5 after the first treatment, eventually leading to cell apoptosis/lysis (Fig. 4b, c, *a*, *b*). The treatment effects were further evaluated by a β-galactosidase senescence assay, and the data showed that senescence cells were detected only in cultures treated with BMP7 alone or combination with TMZ (Fig. 4c, *c*). These observations suggest that protective factors that cause chemoresistance which include anti-senescence, were attenuated when BMP7 signaling was activated, rendering cells more vulnerable, and thereby more sensitive to low-dose TMZ.

### BMP7 modulates molecular properties of GSC associated with treatment resistance

**Fig. 5:**
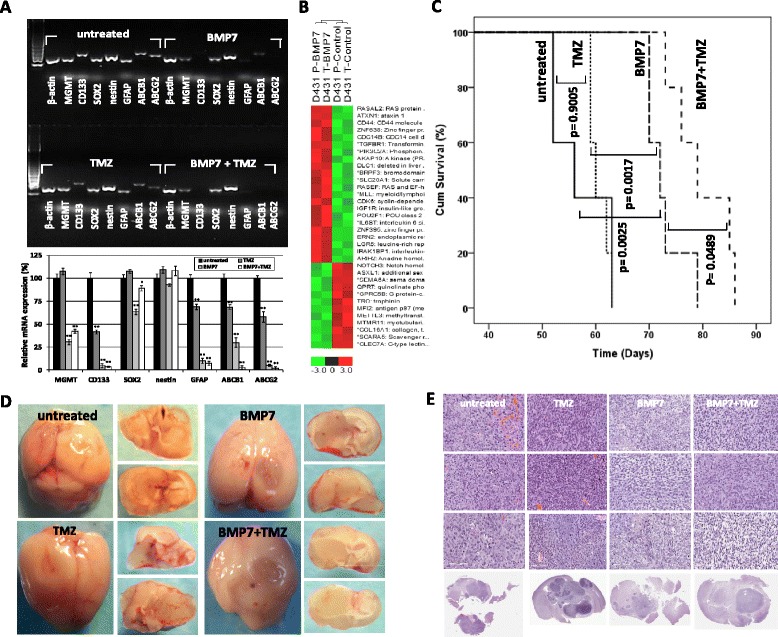
BMP7-induced transcriptional reconfiguration and sensitization of MGMT-expressing GSC to TMZ therapy. **a** Representative results of sqRT-PCR analysis of stemness and TMZ resistance-associated genes in GSC-500 μM TMZ modulated by the indicated treatments. The graph shows the mean values of percentage of mRNA expression levels of indicated genes in D431-500 μM TMZ treated with TMZ (35 μM), BMP7 (100 ng/ml) or their combination relative to those of untreated D431-500 μM TMZ. All values were normalized to those of the internal control gene β-actin, with values of treated D431-500 μM TMZ representing the % relative to that of untreated D431-500 μM TMZ, which was converted to 100 %. Data represent mean values ± SD of triplicate measurements in three independent experiments. *p < 0.05 and **p < 0.001 in relation to GSC-parental. **b** Analyses of gene expression profiles of BMP7-treated D431-parental and D431-500 μM TMZ. Probe set signals on expression array that were ≥ 1.25-fold different in BMP7-treated GSC cultures when compared to untreated GSC cultures were selected (*P* < 0.05). All plots show normalized gene expression values converted into a heatmap. The log2 of the fold difference is indicated by the heatmap scale at the bottom. Each column is an individual GSC samples (P = parental, T = 500 μM TMZ selected). Each row is a single probe set measurement of transcript abundance for an individual gene. The genes and gene functions are listed in the same order from top to bottom as the corresponding table (Table 2). **c** Kaplan-Meier survival curves of untreated, TMZ, BMP7, and combination-treated animals implanted with D431-parental intracranially. The treatment schedules and dosages were described in Methods. P-values were calculated using the Log-rank test. **d** Representative macrophotographic image of glioma xenografts treated with indicated therapy. **e** H-E staining of xenograft tumors from **d**

Previous studies have pointed out that the expression of MGMT/unmethylated MGMT [2, 3, 18], CD133 [22–24], and ATP-binding cassette drug efflux transporters [25–27] are important intrinsic factors contributing to TMZ resistance. We therefore, tested whether we can establish this relationship using MGMT-expressing GSC model. Although BMP7 did not suppress GSC growth effectively in culture as compared to combination treatment (Fig. 4b, c), the BMP7 treatment has induced the loss of CD133 transcripts accompanied by down-regulated MGMT and two drug efflux transporters, ABCB1 (also known as P-glycoprotein or multidrug resistance protein 1) [25, 26] and ABCG2 (breast cancer resistance protein, BCRP) [26, 27] in GSC (Fig. 5a). The expression of SRY (sex determining region Y)-box 2 (SOX2), a marker for neural stem cells, was also decreased but in a lesser degree. Down-regulation of stem cell-associated markers in GSC was more likely to decrease their expression in formerly expressing cells in response to BMP7 treatment, rather than depleting them due to all mRNA samples being extracted 24 h after the first treatment was performed (day 3). Unexpectedly, the expression of glial fibrillary acidic protein (GFAP), a marker of differentiated astrocytes, was not upregulated, but is almost undetectable, while the expression of Nestin remained unchanged. GFAP expressing neural stem cells (NSC) have been reported, although CD133-expressing cells represent a more quiescent NSC-like population [28, 29]. A comparable transcriptional pattern was also seen in GSC treated with BMP7 + TMZ: the expressions of ABCB1 and ABCG2 were almost not detectable. In contrast, the expression of these tested markers remained present in GSC treated with TMZ alone, but the expression levels were lower than those of untreated GSC (Fig. 5a). Moreover, down-regulation of MGMT mRNA levels by BMP7 treatment was not due to the reversal of MGMT promoter methylation status (Additional file 1: Figure S3), suggesting methylation-independent pathways of MGMT expression regulation. In order to obtain more comprehensive knowledge about gene regulation by BMP7 treatment in GSC, we performed an expression microarray analysis to identify genes that were differentially expressed between untreated and BMP7-treated GSC (Fig. 5b, Table 2) in both GSC-parental and GSC-500uM TMZ. DNA microarray demonstrated that BMP7 treatment upregulated a cluster of genes associated with metastasis suppressor (RASAL2, DLC1), EMT/migration/invasion arrest (DLC1, ZNF395), tumor suppressor/cell cycle control (CDC14B, TGFBR1, AKAP10, RASEF, ARIH2), cell differentiation and transcription activation (ATXN1, ZNF638, BRPF3, MLL, IGF1R, IL6ST), insulin signaling/glucose uptake/premature senescence (PIK3C2A, IGF1R), and anti-inflammation/immune response (POU2F1, IRAK1BP1). CD44, a receptor for hyaluronic acid, was determined to be a top 3 upregulated gene; it has been reported that CD44 modulated SMAD1 activation in the BMP7 signaling pathway in chondrocytes [30], and inhibits inflammatory bone loss [31]. Correspondingly, genes down-regulated by BMP7 treatment are mostly associated with EMT/migration/invasion (NOTCH3, TRO, COL16A1), stemness/anti-differentiation/transcriptional repression (NOTCH3, ASXL1), DNA repair/anti-apoptosis/resistance to oxidative stress (QPRT), cell proliferation/tumorigenesis (MFI2, COL16A1), insulin resistance (GPRC5B), angiogenesis (NOTCH3, SEMA6A), and inflammation/immune response (GPRC5B, SCARA5, CLEC7A). The selected genes with distinct expression levels in cells were confirmed by sqRT-PCR analysis (data not shown). These gene profiles therefore, support the view that the molecular properties of EMT and stemness in GSC may be the two key factors triggering drug resistance. The data also suggest that BMP7 possesses the ability of anti-EMT/migration/invasion, induction of tumor stem cell differentiation/senescence, and down-regulation of MGMT and drug efflux transporters, thereby allowing for sensitization of GSC to low-dose TMZ treatment.

**Table 2:** Transcriptional modulation in GSC by BMP7 treatment^a^

Gene	Fold change	P value	Functional involvement
I. Upregulated genes			
RASAL2: RAS protein activator like 2	3.37	0.019083	tumor and metastasis suppressor
ATXN1: ataxin 1	2.59	0.013118	chromatin-binding factor that repress Notch signaling, cell differentiation
CD44: CD44 molecule (Indian blood group)	2.59	0.023871	a receptor for hyaluronic acid; modulates Smad1 activation in the BMP7 signaling
ZNF638: protein 638	2.33	0.030429	adipocyte differentiation
CDC14B: CDC14 cell division cycle 14 homolog B (S. cerevisiae)	2.09	0.027588	cell cycle control
TGFBR1: Transforming growth factor, beta receptor I	1.99	0.015786	cell cycle arrest in epithelial cells; growth inhibition, cell apoptosis
PIK3C2A: Phosphoinositide-3-kinase, class 2, alpha polypeptide	1.63	0.037372	functions in insulin signaling, premature senescence and oxidative apoptosis
AKAP10: A kinase (PRKA) anchor protein 10	1.59	0.024294	control of cell proliferation
DLC1: deleted in liver cancer 1	1.58	0.041191	metastasis suppressor gene, EMT arrest
BRPF3: bromodomain and PHD finger containing, 3	1.44	0.035113	promotes formation of acetyltransferase complexes, stimulates transcription
SLC20A1: Solute carrier family 20 (phosphate transporter), member 1	1.40	0.042665	cellular metabolism, signal transduction, and nucleic acid and lipid synthesis
RASEF: RAS and EF-hand domain containing	1.39	0.045602	tumor suppressor, induction of apoptosis, inhibition of proliferation
MLL: myeloid/lymphoid or mixed-lineage leukemia	1.38	0.024547	regulates neural progenitor proliferation and neuronal and glial differentiation
CDK6: cyclin-dependent kinase 6	1.37	0.047985	augments accumulation of p53, decreases tumor growth when overexpression
IGF1R: insulin-like growth factor 1 receptor	1.34	0.045232	stimulates stem cell differentiation via AKT activation; premature senescence
POU2F1: POU class 2 homeobox 1	1.34	0.032549	anti-inflammation/immune response, cell apoptosis, regulation of NF-kappaB
IL6ST: interleukin 6 signal transducer (gp130, oncostatin M receptor)	1.29	0.022673	stimulates osteoblast differentiation, maintains bone formation
ZNF395: zinc finger protein 395	1.29	0.030385	suppresses cell migration and invasion
ERN2: endoplasmic reticulum to nucleus signaling 2	1.28	0.037335	activation of the unfolded protein response
LGR5: leucine-rich repeat-containing G protein-coupled receptor 5	1.27	0.030355	universal epithelial stem cell marker
IRAK1BP1: interleukin-1 receptor-associated kinase 1 binding protein 1	1.27	0.024291	downregulation of inflammatory cytokines, anti-inflammation, growth inhibition
ARIH2: Ariadne homolog 2 (Drosophila)	1.26	0.032696	inhibits cell proliferation
II. Downregulated genes			
NOTCH3: Notch homolog 3 (Drosophila)	−1.25	0.037102	EMT, maintenance of NSC in an undifferentiated quiescent state, angiogenesis
ASXL1: additional sex combs like 1 (Drosophila)	−1.25	0.016868	suppresses adipogenesis, transcriptional repression
SEMA6A: sema, transmembrane, and cytoplasmic domains, 6A	−1.25	0.041608	vascular development; tumor angiogenesis; neuronal development
QPRT: quinolinate phosphoribosyltransferase	−1.25	0.027582	resistance to oxidative stress; suppression of spontaneous cell death
GPRC5B: G protein-coupled receptor, family C, group 5, member B	−1.26	0.029052	insulin resistance; inflammatory signaling; promotes ERK1/2 activation
TRO: trophinin	−1.26	0.033855	promotes EMT, invasive and metastatic phenotype; tumor formation
MFI2: antigen p97 identified by mAb 133.2 and 96.5	−1.26	0.034824	cell proliferation and tumorigenesis
METTL3: methyltransferase like 3	−1.28	0.046969	plays a role in the efficiency of mRNA splicing and processing
MTMR11: myotubularin related protein 11	−1.35	0.017165	unknown; cisplatin resistance-associated protein
COL16A1: collagen, type XVI, alpha 1	−1.37	0.020495	stimulates tumor proliferation and invasion
SCARA5: Scavenger receptor class A, member 5 (putative)	−1.40	0.024406	innate immune response; inflammatory response
CLEC7A: C-type lectin domain family 7, member A	−1.40	0.009915	innate immune response; inflammatory response; migration and proliferation

*NSC* neural stem cells; *EMT* epithelial-to-mesenchymal transition

^a^Probe set signals on the expression array that were ≥ 1.25-fold different in BMP7 treated GSC cultures (*n* = 2, GSC-parental and GSC-500 μM TMZ) when compared to untreated GSC cultures (*n* = 2, GSC-parental and GSC-500 μM TMZ) were selected

### BMP7 synergizes TMZ in treatment of GSC and extends animal survival

To test whether anti-tumor synergy of combination treatment in culture can be translated to *in vivo* treatment, we performed a proof-of-principle experiment to compare the treatment efficacy of 0.01 % DMSO (untreated), TMZ, BMP7, and combination of BMP7 and TMZ, on preventing tumor initiation and progression (enrichment of resistant clones) in animals inoculated with GSC-parental. We chose D431-parental as the treatment model, because the mice that were injected with D431-parental had the shortest lifespan when compared to those injected with a different line. Moreover, D431-parental contains the highest % of CD133^+^ cells (~35 %) among 3 GSC lines [20]. The administration routes, and dosing schedules are described in Material and Methods. Treatment with TMZ alone did not show a survival benefit (59–63 days) when compared to the untreated animal group (52–63 days) (*p* = 0.9005) (Fig. 5c). Although BMP7 alone did not effectively stop GSC growth in culture, treatment with BMP7 alone demonstrated significantly extended survival of animals (70–79 days) when compared to untreated (*p* = 0.0025) or treated with TMZ alone (*p* = 0.0017) (Fig. 5c). The combination of BMP7 with TMZ further showed a smaller, but significant prolongation of survival (73–86 days) when compared to treatment with BMP7 alone (*p* = 0.0489) (Fig. 5c). Although all mice died of tumor development, a more pronounced vascularity was seen in untreated and TMZ-treated animals when compared to those treated with BMP7 and combination therapy (Fig. 5d, e). Moreover, there was less tumor spreading seen in animals treated with combination therapy (Fig. 5d, e). These results suggest that BMP7 treatment not only can attenuate the tumorigenicity of GSC and delay tumor progression, but also can further synergize with TMZ in enhancing the suppression of the tumor growth thereby providing a greater survival benefit to animals.

## Discussion

In this study, we used molecular profiles of MGMT-expressing GSC that survived high-dose TMZ treatment, to probe defense signatures which could be potential treatment targets for sensitizing GSC to the clinically relevant dose of TMZ. Using this non-biased strategy, we have identified informative gene profiles that are likely to contribute to resistance to high-dose TMZ treatment. These protective stress response profiles are similar to our previous finding in GSC clones that survived radiochemotherapy (RT + TMZ) [13], which expressed molecular and functional characteristics resembling the anti-aging/anti-stress effects of caloric/glucose restriction (GR), by which both insulin-like growth factor 1 (IGF1) and insulin/Akt signaling were reduced [32–34]. The transcription profiles suggested that the stress/drug resistance of GSC-500 μM TMZ is associated with cellular quiescence, EMT/invasiveness, suppressed growth and differentiation, and impaired insulin/Akt signaling. We unintentionally found BMP7 to be a top down-regulated gene in GSC-500 μM TMZ, and thus hypothesized that reduced BMP7 expression/signaling helped them maintain their dedifferentiated state, which prevents them from premature senescence, and renders them more resistant to standard treatment, since it only targets more differentiated/aging cells [35]. Indeed, treatment with BMP7 alone allows for delaying tumor development/progression without TMZ compared to untreatment or treatment with TMZ alone, suggesting that the anti-tumor activity of BMP7 is independent of MGMT status in GSC. This notion is further supported by the finding that BMP7 treatment does not reverse the unmethylation status of GSC. Our data support the view that induction of cell senescence/aging and loss of EMT/migration/invasion properties by BMP7 treatment are likely to contribute to the reduction of tumorigenesis and progression, leading to prolonged animal survival. Moreover, BMP7 treatment down-regulates the expression of MGMT and ATP-binding cassette drug efflux transporters in GSC may provide an additional support mechanism to synergize low-dose TMZ in treatment of GSC. Therefore, BMP7 treatment induces cooperative mechanisms which allow for sensitization of GSC to low-dose TMZ treatment, and extends animal survival. Similar to our finding, a recent study showed that MGMT methylation status does not predict TMZ response in GSC model, and both methylated and unmethylated MGMT bands can be amplified in TMZ-resistant GSC lines [36]. Another study found that some GBM lines resistant to TMZ treatment do not express MGMT protein, but rather exhibit a down-regulation of DNA mismatch repair protein or reduced methylation of LINE-1 repetitive elements (enhances transposon activity) [37]. Therefore, we believe that down-regulation of MGMT expression by BMP7 is not the sole mechanism allowing for GSC responding to low-dose TMZ treatment.

BMP7 is a member of the transforming growth factor-β (TGF-β) superfamily of growth factors. The binding of BMP7 to its receptors, BMP type 1 and type 2 receptors (BMPR1/2), induces the phosphorylation of intracellular SMAD1/5/8, which can block the nuclear translocation of phosphorylated SMADs 2/3 induced by TGF-β, which in turn results in suppressed TGF-β signaling. BMP7 plays a pivotal role in the osteoblast differentiation/bone formation, kidney development, and promoting brown adipocyte differentiation [38–40]. Recently, BMP7 has been implicated in regulation of cancer pathogenesis and metastasis, possibly due to its ability to counteract TGF-β-induced, SMAD3-dependent EMT [41, 42]. Reduced levels of BMP7 in primary breast and lung cancer tissues are significantly associated with the formation of clinically overt bone metastases for breast cancer patients and lymph node metastasis for lung cancer patients [43, 44]. Down-regulated BMP7 expression was also determined in primary human prostate cancer tissue when compared with normal prostate luminal epithelium [45]. The animal studies further demonstrated that BMP7 treatment significantly inhibited internal bone growth of breast cancer cells and prostate cancer bone metastases, suggesting that reduced BMP7 signaling in tumor cells may enhance tumorigenesis and EMT with the development of metastatic properties via enhanced capacity for cell migration and invasion [43, 45]. Our previous [13] and current data support the view that EMT linked with dedifferentiation/stemness are possibly underlying treatment resistance of GSC, which can be overridden by augmenting BMP7 signaling. The removal of factors associated with stemness or anti-stress properties in GSC by BMP7 treatment is evident by demonstrating the significantly downregulation of mRNA levels of CD133, MGMT, and efflux transporters with concomitant induction of cell senescence and susceptibility to low-dose TMZ treatment. A previous study has shown that induction of EMT in mammary epithelial cells by exposure to TGF-β1 or overexpression of Snail or Twist (EMT-inducing transcription factors), can generate cells with stem cell properties [46]. Another study also found that ZEB1, an EMT activator, represses expression of stemness-inhibiting microRNA [47]. Likewise, we previously found that primary glioblastoma possess molecular properties of mesenchymal stem cells (MSC) and express cellular and molecular markers that have been implicated in EMT/myofibroblastic phenotype [48]. In particular, we found primary glioblastoma tumor cells express CD105 (endoglin), which is an MSC surface marker and a component of the TGF-β receptor complex that binds to TGF-β1 and TGF-β3, and modulates TGF-β signaling [48], thus suggesting a direct link between TGF-β-induced EMT and the gain of stem cell properties in glioblastoma and GSC. TGF-β1 promotes hematopoietic stem cell quiescence by downregulating Akt activity and upregulating FOXO3 activity [49]. Likewise, we also found that GSC clones sensitive to radiochemotherapy (RT + TMZ) exhibited activated Akt activity with increased glucose usage, whereas resistant clones expressed upregulated CD133, SOX2 and MGMT, with reduced Akt activity and increased AMPK-SIRT1-FOXO Axis and favored the fatty-acid oxidation pathway for their energy source [13]. In this study, we also found that treatment with BMP7, an antagonist of TGF-β system, downregulated EMT and stemness transcription program accompanied by upregulation of genes associated with insulin signaling/AKT and cell senescence, suggesting that BMP7 modulates stemness and EMT may be involved in a metabolic switch, which led to increased drug sensitivity. Similarly, a recent study showed that BMP2 can sensitize glioblastoma-stem-like cells to TMZ (500 μM) by downregulating both hypoxia-inducible factor-1α (HIF-1α) and MGMT [50]. The study also demonstrated a direct binding of HIFs to the MGMT promoter under hypoxia, and the treatment with BMP2 can abrogate HIF-1a binding to MGMT promoter [50]. The hypoxia/HIF-dependent promotion of the stemness/cellular quiescence and EMT in cancer progression has been well addressed and reviewed [51–53]. Correspondingly, it was reported that ABC efflux transporters contain several binding sites for EMT-inducing transcription factors, and overexpression of Twist, Snail, and FOXC2 can increase promoter activity of ABC efflux transporters by binds directly to the E-box elements of ABC efflux transporters [54]. These findings therefore support the notion that BMP7-mediated downregulation of CD133, MGMT and efflux transporters accompanied with increase sensitivity to TMZ may be achieved via the removal of factors that promote EMT and stemness properties in GSC.

We previously reported that GSC clones surviving radiochemotherapy (RT + TMZ), or purified CD133^+^ GSC, expressed upregulated angiogenesis- and EMT-associated genes [13, 17]. In this study, we further found that BMP7 treatment markedly upregulates genes associated with cell differentiation and senescence while downregulates genes associated with stemness properties in GSC, including CD133 expression, suggesting that the expression of CD133 may be an indication of both stemness and drug resistance in GSC [22–24]. Recent reports indicated that CD133 is a marker of bioenergetic stress in hypoxic human glioma [55] and that activation of hypoxia/HIF-1alpha enhanced the self-renewal activity of CD133^+^ GSC and inhibited the induction of CSC differentiation [56]. Moreover, the expression of CD133 facilitates EMT [57], whereas CD133 silencing inhibits stemness properties and enhances chemoradiosensitivity of tumor stem cells [58]. In this study, we identified several reported EMT inducers as molecular signatures of GSC-500 μM TMZ, including MCAM, FN1, and MALAT1 [59–61]. Correspondingly, NOTCH3, which both gates neural stem cell activation [62] and contributes to TGF-β-induced EMT [63], was detected as a downregulated gene by BMP7 treatment. Meanwhile, a NOTCH signaling repressor, ATXN1 [64], was identified as an upregulated gene by BMP7 treatment. Thus, maintaining the low activity of BMP7 signaling in GSC may be required for sustaining EMT, stemness, and multidrug-resistant phenotype. Our finding is in agreement with that of others showing that BMP7 release from endogenous neural precursor cells can induce tumor stem cell differentiation and reduce the ability for tumor initiation, therefore, providing a protective action in animals [65]. Likewise, treatment with a BMP7 variant suppressed the tumorigenicity of stem-like glioblastoma cells and reduced angiogenesis and brain invasion [66]. The optical imaging study further provided direct evidence of BMP7-induced cell cycle arrest in glioma model [67]. Notably, it was reported that BMP7-induced senescence of prostate cancer stem-like cells is reversible; withdrawal of BMP7 treatment restarted growth of these cells in bone [68]. Therefore, combination with chemotherapy would provide extended survival time by eliminating the cells, preventing them from regrowing back. Genetic approaches for elucidation of mechanisms by which BMP7 downregulates MGMT, ABC efflux transporters, stemness, and EMT would further facilitate our understanding the process and ability to identify new treatment targets and strategies for preventing treatment resistance and tumor recurrence.

## Conclusion

The data presented in this study showed that BMP7 treatment can sensitize MGMT-expressing GSC to the clinically relevant dose of TMZ both *in vitro* and *in vivo*. The gene profiles pointed out that BMP7-mediated TMZ sensitization in GSC may be associated with reprogramming of transcriptional profiles, particularly the downregulation of genes which contributed to EMT/migration/invasion, stemness, and drug resistance. Our data therefore, suggest a potential therapeutic utility of BMP7, a neuroprotective agent in cerebral hypoxia/ischemia [69, 70], combined with TMZ, for treating newly diagnosed glioblastoma or recurrent diseases exhibiting unmethylated MGMT.

## Methods

### Cell cultures

Glioblastoma stem cell (GSC) cultures used in this study were established from glioblastoma tumor tissues derived from patients who underwent surgery at Ronald Reagan UCLA Medical Center. All samples collected were under patients’ written consent, and were approved by the UCLA Institutional Review Board. The tumors were enzyme-digested and washed, followed by red blood cell (RBC) lysis of the pellet. The primary cells were cultured in a serum-free stem cell culture medium consisting of DMEM/Ham’s F-12 (Mediatech, Manassas, VA), 20 ng/ml human recombinant epidermal growth factor (EGF, Sigma-Aldrich, St. Louis, MO), 20 ng/ml basic fibroblast growth factor (FGF, Chemicon, Billerica, MA), 10 ng/ml leukemia inhibitory factor (LIF, Chemicon), and B27 without vitamin A (Invitrogen, Carlsbad, CA). The tumor spheres were dissociated and replated at clonal density and continually passaged until the clonogenic cells were stably maintained. As previously described [13, 17], the D431 GSC culture line was classified as mesenchymal subtype whereas S496 and E445 culture lines were classified as proneural (PN) subtype. All three tumorigenic GSC cultures contained CD133^+^ cells (39.5 %, 9.6 % and 1.5 % respectively), exhibited wild-type IDH1/IDH2 and unmethylated MGMT promoter and expressed MGMT transcripts (10, 14, Additional file 1: Figure S3).

### Isolation of clonogenic GSC resistant to high-dose TMZ

GSC cultures were seeded at clonal density overnight, and treated with 500 μM TMZ the next day. Fresh media was replaced every three days. TMZ treatment was repeated on day 7 after the first treatment to ensure that clonogenic survivors are truly resistant to 500 μM TMZ. Clonogenic cells that have survived were isolated, expanded, and designated as GSC-500TMZ-C1 (treatment cycle 1). Established GSC-500TMZ-C1 cultures were then reseeded and re-treated with 500 μM TMZ twice (day 1 and day 7), and the stable clonogenic cells continued to grow were harvested and designated as GSC-500TMZ-C2. In order to maintain resistant clones in the long-term cultures, cells were re-exposed to 500 μM TMZ treatment after thawing or prior to using in experiments.

### Cell proliferation

The effects of TMZ or siRNA treatment on GSC growth were determined by a 3-(4, 5-dimethylthiazol-2-yl)-5-(3-carboxymethoxyphenyl)-2-(4-sulfophenyl)-2H-tetrazolium (MTS/PMS) colorimetric assay according to the manufacturer’s instructions (Promega).

Cells were seeded in 96-well tissue culture plates at a density of 6,000–10,000 cells per well per 100 μL stem cell media in triplicate in the presence or absence of indicated treatment. Cells were incubated for 48–72 h, and absorbance was measured at 490 nm after a 4-hour incubation with MTS/PMS reagents.

### Cell apoptosis

Cell apoptosis in BMP7- and TMZ-treated GSC was determined by Apo-ONE® Homogeneous Caspase-3/7 Assay kit (Promega), according to the manufacturer's protocol. One hundred microliters Homogeneous Caspase-3/7 Reagent was added to each well followed by 2 min mixing on a plate shaker. Plates were incubated at room temperature for 2 h. Fluorescence intensity was measured with a fluorescence microplate reader (Synergy HT, BioTek) at the excitation and emission wavelengths of 485 nm and 528 nm respectively. Multiple readings were taken at one-hr intervals. Data are expressed as the relative fluorescence units (RFU).

### Cell-cycle distribution

2–5 × 10^5^ dissociated cells were washed twice with cold PBS. Cell pellets were resuspended in 1 ml propidium iodide hypotonic DNA staining buffer (50 mg/ml propidium iodide, 0.1 % Triton X-100, and 0.1 % sodium citrate in PBS) and mix well. Samples were kept in 4 °C away from light for a maximum of 1 h before acquisition on the flow cytometer for cell cycle analysis (FACScan, Becton Dickinson), which used DNA content as a measure of progression in cell cycle and as means of detecting apoptotic cells. Apoptotic cells with degraded DNA were detected as a hypodiploid or "sub-G1" peak in a DNA histogram.

### Microarray procedures, data analysis and gene annotation

Molecular profiling and analysis were performed as described [17]. Briefly, cDNA was generated and converted to cRNA probes using standard Affymetrix protocols and hybridized to Affymetrix GeneChip U133 Plus 2.0 Array. The chips were scanned using the GeneArray scanner (Affymetrix). The CEL files generated by the Affymetrix Microarray Suite version 5.0 were converted into DCP files using the DNA-Chip Analyzer (dChip/2008; http://www.hsph.harvard.edu/cli/complab/dchip/). The DCP files were globally normalized, and gene expression values were generated using the dChip implementation of perfect-match minus mismatch model-based expression index. All group comparisons were performed in dChip. Functional annotation of individual genes was obtained from NCBI/Entrez Gene (http://www.ncbi.nlm.nih.gov/sites/entrez), the published literature in PubMed Central (NCBI/PubMed), GeneCards (http://www.genecards.org/), and Protein knowledge base (UniProtKB) (http://beta.uniprot.org/). All microarray CEL files analyzed in this study are accessible from the Gene Expression Omnibus (GEO) (Series Accession number: GSE68071).

### Semi-quantitative reverse transcriptase polymerase chain reaction (sqRT-PCR) analysis

Cells were subjected to total RNA extraction using RNeasy kit (Qiagen, Valencia, CA). Two micrograms of total RNA from each sample were reverse transcribed to cDNA using a TaqMan RT Reagent Kit (Applied Biosystems). Thirty cycles of PCR amplification was performed on an Eppendorf gradient thermocycler, using 5 μL cDNA, SYBR Green PCR Core Reagents (Applied Biosystems) and gene-specific primers (Invitrogen). The signal intensity of each specific gene was quantified using Image Lab™ Software (Gel Doc™ EZ System, Bio-Rad). The band intensity of each sample was normalized to the corresponding β-actin band intensity in order to obtain the relative level of gene expression. The primer sequences and expected sizes of amplified PCR products are described in Additional file 1: Table S1.

### Western blot analysis

30 μg protein from each sample were separated on 4–20 % gradient SDS-PAGE (Bio-Rad) and transferred onto a PVDF membrane. The blots were incubated with phospho-Smad1 (Ser463/465)/ Smad5 (Ser463/465)/Smad9 (Smad8) (Ser465/467) antibody (Cell Signaling) for overnight at 4 °C. Anti-β-actin (Cell Signaling) was used as internal control. The blots were washed and incubated with horseradish peroxidase-conjugated anti-rabbit IgG for 1 h. After washing, blots were incubated with Pierce Supersignal ECL substrate, and exposed to X-ray films.

### siRNA transfection

A reverse transfection protocol was done to deliver non-silencing negative control siRNA (scrambled siRNA) (Ambion), glyceraldehyde-3-phosphate dehydrogenase (GAPDH) or gene-specific siRNA (Ambion), into GSCs as previously described [13]. The transfection efficiency and cellular toxicity due to transfection were monitored using KDalert™ GAPDH Assay Kit (Invitrogen). Briefly, a transfection complex was prepared by diluting siRNA in 10 μl OPTI-MEMI (Invitrogen) then mixing with 10 μL OPTI-MEMI containing 0.3 uL Lipofectamine RNAi-MAX transfection reagent (Invitrogen). The siRNA transfectant was then added into each well in a 96-well plate followed by seeding 6000–9000 cells in 100 μL media to give a final siRNA concentration of 30 nM in each well. Targeted gene silencing was determined 72 h after transfection by sqRT-PCR, using a Power SYBRH Green Cells-to-CTTM Kit (Ambion).

### Methylation-specific PCR (MSP) analysis

Genomic DNA was subjected to bisulfite treatment using the EZ DNA Methylation-Gold™ Kit (Zymo Research) following the manufacturer's instructions. The promoter MGMT-MSP was performed using a two-step nested approach to amplify both methylated and unmethylated MGMT separately as described previously [71]. Total human genomic DNA methylated by bacterial DNA methyltransferase and whole genome amplified DNA were used as positive and negative controls for methylated alleles of MGMT, respectively. The PCR products were resolved on 4 % low melting point agarose gels.

### Intracranial tumor formation, BMP7 treatment, and histopathologic analysis

To test the tumorigenic capacity of GSC, 10^5^ viable cells in a volume of 3 μl culture media were engrafted intracranially into anesthetized NOD (CB17-Prkdcscid/J) mice (5 weeks old; 15–16 g). Mice were then maintained until neurological signs were observed, at which point they were sacrificed. The brains were removed, fixed in 4 % formalin, paraffin-embedded, and sectioned. Histopathologic analyses were done on paraffin slides stained with hematoxylin-eosin (H-E) staining per standard technique. To test whether BMP7 can sensitize TMZ for treatment of GSC *in vivo*, mice were randomly divided into four groups (5 mice per group) and each group was received the following treatments: group 1, 0.01 % DMSO; group 2, TMZ; group3, BMP7; group 4, BMP7 + TMZ. GSC cells (10^5^/3 μl/animal) were intracranially injected followed by local delivery of vehicle (0.01 % DMSO in 100 μl H_2_O) or TMZ (100 μl of 35 μM TMZ) for group 1 and group 2 mice or BMP7 (10 ng in 100 μl water) for group 3 and group 4 mice using an osmotic minipump (Alzet, USA, model 1007D). The sterile pump was implanted subcutaneously (s.c.) onto the back of animals, and the drug solutions were delivered through the cannula, which was placed through a small skull burr hole onto the pial surface where tumor cells were implanted. On day 7 after local treatment, the mice of group 2 were continuously treated with TMZ (66 mg/kg) via oral gavage daily for 5 days, the mice of group 3 received intraperitoneal injection of BMP7 (2 μg/day) for 5 days and mice group 4 received both BMP7 (morning) and TMZ (afternoon) for 5 days. Animals were maintained until neurological signs were observed. Immediately after sacrifice, the brains were removed and subjected to histopathological analysis. All animal experiments in this study were under a protocol approved by the UCLA Institutional Animal Research Committee.

### Statistical analysis

Each experiment was set up in triplicate and repeated at least twice. Data were expressed as means ± SD and analyzed using 1-way ANOVA tests, depending on homogeneity of variances. Cumulative survival probabilities were calculated using the Kaplan-Meier method. The log-rank test was used to compare survival across groups. All p-values were 2-sided, and those lower than 0.05 were considered significant. SPSS v19.0 for Windows software was used for all statistical analysis.

## Additional file

Additional file 1: **Supplementary Materials.** (DOCX 1426 kb)
