# Supplementary material for: Bone morphogenetic protein 7 sensitizes O6-methylguanine methyltransferase expressing-glioblastoma stem cells to clinically relevant dose of temozolomide
Source: Mol Cancer. 2015 Nov 6;14:189. doi: 10.1186/s12943-015-0459-1 (PMC4636799; doi:10.1186/s12943-015-0459-1)
Supplement: Supplementary file 1 — Supplementary Materials. (DOCX 1426 kb) [file 12943_2015_459_MOESM1_ESM.docx]

**Supplementary Figure 1**. Analyses of distinct gene expression profiles of GSC-parental and GSC-500µM TMZ. A. Probe set signals on expression array that were ≥ 1.5-fold different in GSC-500 µM TMZ (n=3 patients, 6 samples) when compared to GSC-parental (n=3 patients, duplicate samples) by a pairwise t-test (P < 0.05), were selected. All plots show normalized gene expression values converted into a heatmap. The log2 of the fold difference is indicated by the heatmap scale at the bottom. Each column is an individual GSC sample. Each row is a single probe set measurement of transcript abundance for an individual gene. The genes are listed in the same order from top to bottom as the corresponding Table 1 described in the text. B. The distinct gene expression in GSC-parental and GSC-500µM TMZ was confirmed by sqRT-PCR analysis.

**A**

**B**

**Supplementary Figure 2**. Inhibition of GSC-500µM TMZ self-renewing capacity by knockdown of defense signatures of GSC-500µM TMZ. A. E445-500µM TMZ were treated with siRNA targeting indicated defense signatures of GSC-500µM TMZ in the presence or absence of 35 μM TMZ. Photos were taken 3 days after transfection. B. S496-500µM TMZ and E445-500µM TMZ were treated with siRNA targeting a representative signature, NNMT, combined with and without TMZ. Photos were taken 7 days after transfection.

**Supplementary Figure 3**. Methylation-specific PCR (MSP) of MGMT in GSC. Methylation status of MGMT promoter in GSC was determined by a nested methylation-specific PCR assay. Cells were treated without (A) or with BMP7 (B) for 5 days. GSC names are indicated above the bands. Bisulphite-modified DNA was amplified with primer sets specific for unmethylated and methylated DNA. Total human genomic DNA (gDNA) methylated by bacterial DNA methyltransferase was used as positive control for the methylated MGMT promoter. Total human gDNA amplified by using whole genome amplification kit was used as negative control. In most GSC lines/clones, both U and M products were generated, suggesting that the samples contain both methylated and unmethylated cell population. U= Unmethylated 93-bp PCR product, M= Methylated 81-bp PCR product.

**Supplementary Table 1. Primer sequences and product sizes for semi-qtRT-PCR analysis**

**Beta-ACTB:**

LEFT PRIMER ACTCAGGATTTAAAAACTGGAACG

RIGHT PRIMER GACTTCCTGTAACAATGCATCTCAT

PRODUCT SIZE: 152

**MB2:**

LEFT PRIMER GAGCAAATCTTTGGTCAAGTTAAAA

RIGHT PRIMER ATACTCAATCTTGAGGGAAAAATCC

PRODUCT SIZE: 244

**GRP:**

LEFT PRIMER AGAGAAAAACAAAACCCCTAAGAGA

RIGHT PRIMER ACAGCAAACAAGTTTAAACCAGAAG

PRODUCT SIZE: 163

**NNMT:**

LEFT PRIMER CTACTACATGATTGGTGAGCAGAAG

RIGHT PRIMER AGGAATTGCTTTAATTGAGGTCAC

PRODUCT SIZE: 219

**SYTL2:**

LEFT PRIMER CAAACAGTTAAAACAAGCCTCAAAT

RIGHT PRIMER TGTGACTACTCTTATGCTGCTTTCA

PRODUCT SIZE: 202

**PPP1R14C:**

LEFT PRIMER AATAGGTGAGAGTAGAAACCCTTCC

RIGHT PRIMER TACATTTTCAAAAGCTTGCCAGTAT

PRODUCT SIZE: 203

**FAM46A:**

LEFT PRIMER CCTTGCAGAACGGAGGAG

RIGHT PRIMER TCCTTTTCTGCCTGTGACG

PRODUCT SIZE: 156

**RUNDC3B:**

LEFT PRIMER GCTGCTAAATGCAGATTTAAACATT

RIGHT PRIMER GAGGGTGAACAATAAACACTACCAC

PRODUCT SIZE: 209

**GAPDH:**

LEFT PRIMER ATTTCCATTGATGACAAGCTTCC

RIGHT PRIMER CTGCTTTTAACTCTGGTAAAGTGGA

PRODUCT SIZE: 155

**BMPR2:**

LEFT PRIMER GGATGCCACTATGAAAGCTGA

RIGHT PRIMER AACGCACCATTTTACAGCAA

PRODUCT SIZE: 179

**BMP7:**

LEFT PRIMER AGATTATTTATTTCCATCCAAAGCTC

RIGHT PRIMER ACAGAATTTTCACTAAGGACTGCTC

PRODUCT SIZE: 178
